# Cost-effectiveness analysis of vaborem for the treatment of carbapenem-resistant Enterobacteriaceae-*Klebsiella pneumoniae* carbapenemase (CRE-KPC) infections in the UK

**DOI:** 10.1007/s10198-021-01375-0

**Published:** 2021-09-21

**Authors:** Ioanna Vlachaki, Daniela Zinzi, Edel Falla, Theo Mantopoulos, Holly Guy, Jasimran Jandu, Andrew Dodgson

**Affiliations:** 1 Menarini Ricerche SpA, Athens, Greece; 2 Menarini Ricerche SpA, Florence, Italy; 3 Real World Solutions, IQVIA Ltd, 37 North Wharf Road, London, W21AF UK; 4 Real World Solutions, IQVIA Ltd, Athens, Greece; 5 FIECON Ltd, St Albans, UK; 6 Public Health England, Manchester, UK

**Keywords:** Meropenem-vaborbactam, Carbapenem-resistant Enterobacteriaceae—*Klebsiella pneumoniae* carbapenemase, Cost-effectiveness, Best available treatment, I11

## Abstract

**Objective:**

The study objective of this analysis was to determine the cost-effectiveness of vaborem (meropenem-vaborbactam) compared to the best available therapy (BAT) in adult patients with carbapenem-resistant Enterobacteriaceae—*Klebsiella pneumoniae* carbapenemase (CRE-KPC) infections from the perspective of the UK National Health Service (NHS) and Personal Social Services (PSS).

**Methods:**

A decision tree model was developed to conduct a cost-effectiveness analysis for Vaborem compared to BAT in CRE-KPC patients over a 5 year time horizon. The model structure for Vaborem simulated the clinical pathway of patients with a confirmed CRE-KPC infection. Model inputs for clinical effectiveness were sourced from the TANGO II trial, and published literature. Costs, resource use and utility values associated with CRE-KPC infections in the UK were sourced from the British National Formulary, NHS reference costs and published sources.

**Results:**

Over a 5 year time horizon, Vaborem use increased total costs by £5165 and increased quality-adjusted life years (QALYs) by 0.366, resulting in an incremental cost-effectiveness ratio (ICER) of £14,113 per QALY gained. The ICER was most sensitive to the probability of discharge to long-term care (LTC), the annual cost of LTC and the utility of discharge to home. At thresholds of £20,000/QALY and £30,000/QALY, the probability of Vaborem being cost-effective compared to BAT was 79.85% and 94.93%, respectively.

**Conclusion:**

Due to a limited cost impact and increase in patient quality of life, vaborem can be considered as a cost-effective treatment option compared to BAT for adult patients with CRE-KPC infections in the UK.

**Supplementary Information:**

The online version contains supplementary material available at 10.1007/s10198-021-01375-0.

## Introduction

Carbapenem-resistant Enterobacteriaceae (recently classified as *Enterobacterales*) (CRE) are among the most critical group of multidrug-resistant bacteria worldwide. In an effort to aid the prioritisation of research and development of new antibiotics, the World Health Organization (WHO) has listed CRE as a critical priority pathogen due to its increasing incidence and high mortality and morbidity worldwide [1]. Antibiotic-resistant bacteria pose a global threat to human health and each year they are responsible for about 33,000 deaths estimated to cost approximately 1.1 billion Euros to the healthcare systems of the European Economic Area (EEA) countries [2]. In recent years, CRE incidence has increased significantly and is the fastest-growing drug-resistant organism in Europe in terms of morbidity and as mortality [2]. Globally, *Klebsiella pneumoniae* carbapenemase (KPC) is the most prevalent and widespread of the five major carbapenemases produced by CRE, rendering it a clinically relevant area of research [3, 4]. Approximately 40 different KPC variants have been described, with KPC-2 and KPC-3 being the most frequently reported ones globally [3].

CRE cause severe infections often acquired in the healthcare setting, including bloodstream infections, pneumonia, complicated abdominal and urinary tract infections which result in longer hospital stays, higher mortality rates and increased healthcare costs compared to infections caused by non-CRE-pathogens [5, 6]. Carbapenem resistance was first observed in England in 2003 and since then, the gradual increase in the incidence of KPC has accounted for approximately 11% of all cases referred to the Antimicrobial Resistance and Healthcare-Associated Infections (AMRHAI) Reference Unit in 2018 [7]. In the UK, KPC poses a significant management challenge for hospitals, particularly in north-west England where KPC-2 is the predominantly (> 95%) disseminated KPC producer [8]. Cost estimates of CRE outbreaks have been exceptionally high in the UK; a cost evaluation of CRE outbreak occurring in five hospitals in the UK showed that prolonged CRE outbreaks over 10 months are associated with costs exceeding £1 million and another outbreak in north-west England was estimated to cost £5 million to the NHS [7, 9, 10].

The rise of CRE infections and lack of safe and effective treatment options for pathogens resistant to carbapenems, has emphasised the need for new antibiotics in recent times. Colistin and tigecycline are still considered as options for the treatment of CRE infection, despite colistin being associated with toxicity concerns such as nephrotoxicity and neurotoxicity. This is also the case with less common agents considered for treatment, including polymyxin B and aminoglycosides. These agents have also been affected by drug resistance, necessitating dose adjustment to treat CRE-KPC infections and in turn resulting in even greater toxicity and poor clinical outcomes [11]. Meropenem combination therapies have shown lower mortality and higher treatment success rates compared to monotherapy in critically ill patients; however, these combinations have never been evaluated in a prospective randomised controlled trial [12–14].

Novel drugs recently approved by the European Medicines Agency (EMA) and the US Food and Drug Administration (FDA) include ceftazidime-avibactam, ceftolozane-tazobactam, meropenem-vaborbactam, eravacycline, imipenem/cilastatin-relebactam and cefiderocol [15–21]. Plazomicin is approved only by FDA [22]. Novel drug discovery for the treatment of multidrug-resistant Gram-negative infections has targeted combinations of a β-lactam molecule with a β-lactamase inhibitor against carbapenemases. Vaborem (meropenem-vaborbactam) was developed in response to a high unmet need focusing specifically on managing CRE-KPC infections due to limited treatment options. The discovery of a novel boron-based β-lactamase inhibitor with a spectrum of inhibition and pharmacological properties that would complement a carbapenem led to the development of vaborbactam. Meropenem, a widely used drug, was selected as the best in class carbapenem, and to exploit its established pharmacokinetic-pharmacodynamic (PK-PD) properties, an optimised dosage regimen with a higher dose and prolonged infusion was proposed for the fixed-dose combination. Vaborbactam achieved targeted inhibition of Class A (KPC enzymes) and C serine carbapenemases with a wide safety margin and no additive toxicity or effects on the well-known safety and tolerability of meropenem. Robust non-clinical and clinical PK-PD assessments, along with the well-known meropenem profile and the recognised high unmet need, led to the development of Vaborem as monotherapy in difficult-to-treat patients (TANGO I clinical trial) and in subjects with severe multidrug-resistant infections due to CRE-KPC (TANGO II clinical trial) [23, 24].

The TANGO II trial was the first prospective, randomised study comparing Vaborem to a pool of antibiotics, selected among the standards of care (SoC) used alone or in combination, as best available therapy (BAT) for CRE infections. This trial with a pathogen focus was specifically designed for CRE-KPC infections, enrolling difficult to recruit patients, usually excluded to participate in this kind of study. Data available from clinical trials and surveillance studies have demonstrated that Vaborem monotherapy is associated with higher clinical cure, lower mortality rates and lower incidence of nephrotoxicity compared to BAT in KPC producing CRE infections [25]. However, there are no existing UK cost-effectiveness analyses of Vaborem and this is the first economic evaluation to use data from TANGO II trial. A cost-effectiveness evaluation was conducted to determine incremental costs and quality-adjusted life years (QALYs) associated with Vaborem compared to BAT in adult patients with CRE-KPC infections from the perspective of the National Health Service (NHS) and Personal Social Services (PSS) UK.

## Methods

### Population

The study population comprised of adult patients with confirmed CRE-KPC associated infections. The CRE-KPC infections included in the patient population were complicated urinary tract infection {cUTI; including acute pyelonephritis [AP]}, complicated intra-abdominal infection (cIAI), hospital-acquired pneumonia {HAP; including ventilator-associated pneumonia [VAP]}, or bacteraemia. The study reflected the primary analysis population of the TANGO II study, representing patients with microbiologically confirmed CRE-KPC infections in the modified intent-to-treat (mCRE-MITT) population [24].

### Intervention and comparators

In line with the TANGO II study and ratified by UK clinicians, the economic model considered Vaborem as the intervention (as monotherapy) and BAT as the main SoC comparator as in the UK clinical practice [26]. BAT included (alone or in combination): a carbapenem, aminoglycoside, polymyxin B, colistin, tigecycline or ceftazidime-avibactam (monotherapy only) (Online Resource 1: Table 3) [24]. Dosing and administration for Vaborem and BAT were obtained from the British National formulary (BNF) [27]. In the TANGO II mCRE-MITT population, of those in the comparator arm, 67% received combination therapy (mCRE-MITT), usually including a carbapenem agent.

### Model structure

**Fig. 1:**
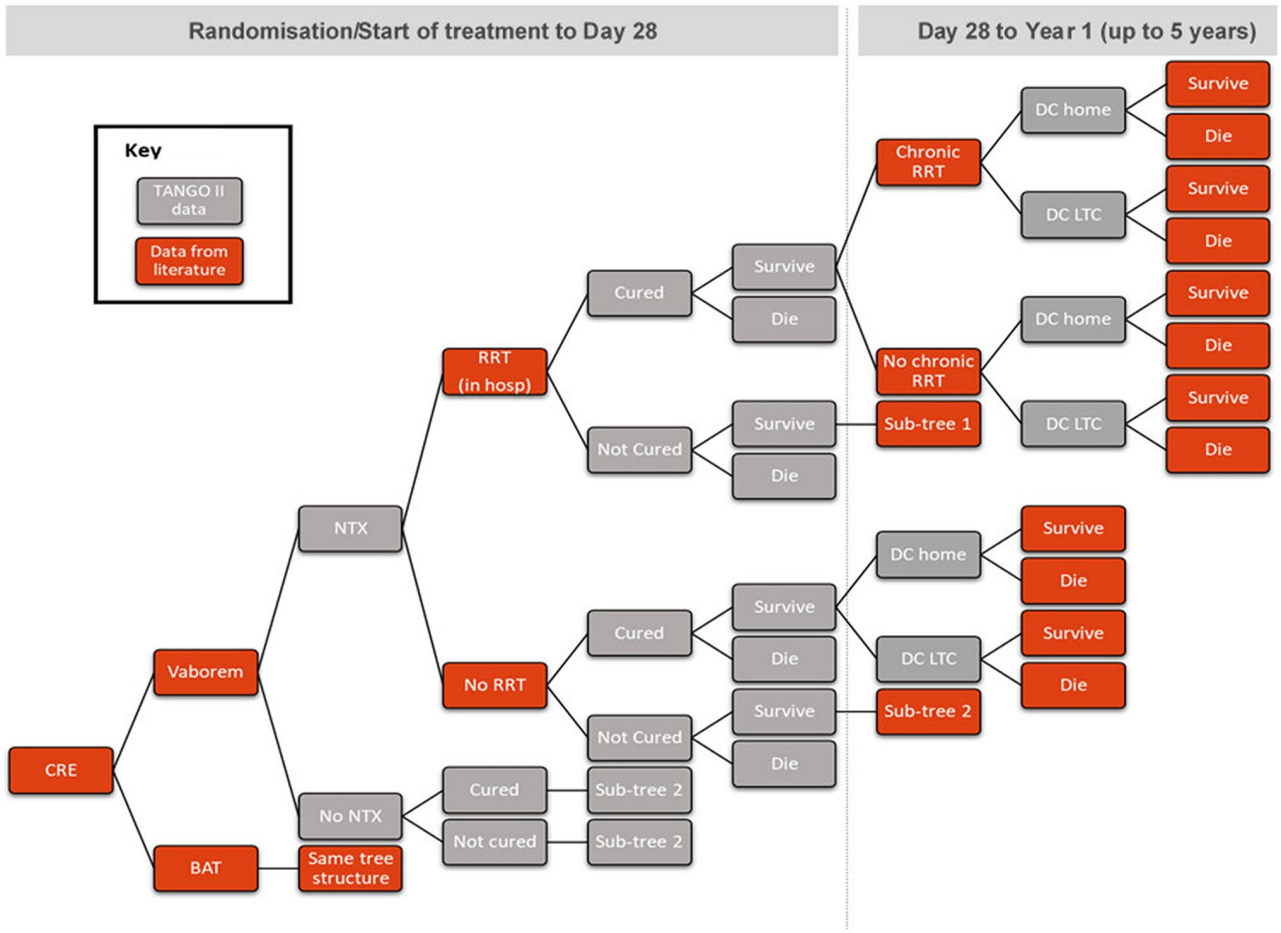
Decision tree model structure. Decision tree model structure simulates the clinical pathway followed by patients with CRE-KPC infections. Data sourced form TANGO II and published literature are indicated by the grey and orange boxes, respectively. *BAT* best available therapy; *CRE* carbapenem-resistant Enterobacteriaceae; *DC* discharged; *LTC* long-term care; *NTX* nephrotoxicity; *RRT* renal replacement therapy; *VAB* Vaborem

A decision tree model was developed in Microsoft Excel^®^ to conduct cost-effectiveness analysis (CEA) with a model structure that simulated the clinical pathway followed by patients with a confirmed CRE-KPC infection. A decision tree model was chosen as it is particularly suited to modelling acute care decision problems such as bacterial infections (Fig. 1).

Two cohorts, each with 1000 patients with a confirmed CRE-KPC infection entered the decision tree and received either Vaborem or BAT, with treatment commencing on day 1 until day 7–14. Following drug initiation, the probability of patients developing antibiotic-induced nephrotoxicity was sourced from the TANGO II trial [28]. Depending on the severity of the nephrotoxicity, patients may require short-term renal replacement therapy (RRT) while hospitalised, for which the probability was sourced from published literature [29]. Treatment can last 6 days for some patients and continue for a longer duration, depending on the individual’s requirement and the extent of renal damage. That is, patients continue treatment if they are not cured or require chronic RRT.

Following this, patients underwent a test of cure assessment {7 days [± 2 days] after the end of treatment} between day 12 and day 23. Patients were either cured or not cured at this stage. Subsequently, patients faced a probability of survival at day 28. The probability of cure and the probability of all-cause mortality at day 28 were sourced directly from the TANGO II trial [24].

The probability of all-cause mortality, sourced from UK life tables from day 28 to Year 5 was adjusted by the baseline demographics of patients in the primary analysis population, underlying comorbidities using the Charlson comorbidity index (CCI) and hazard ratios [30]. Those patients on chronic RRT faced an increased risk of mortality.

Patients who survived CRE-KPC infection, in either treatment arm, then enter a clinical pathway which follows events from 28 days onwards and were modelled for 5 years. In the longer-term, patients with nephrotoxicity were subject to irreversible damage which required chronic RRT for which the probability was sourced from published literature (Online Resource 1: Fig. 1) [29]. The clinical pathway from either node indicated patients being discharged home or discharged to long-term care (LTC), for which the probabilities were sourced from an analysis of patient-level data from the TANGO II trial [28]. All mortality probabilities were sourced from published literature. Patients who do not experience nephrotoxicity or receive RRT also followed this decision tree structure from the point of discharge (Online Resource 1: Fig. 2).

The time horizon of this study was five years, which is consistent with other CEA studies as identified from an economic systematic literature review (SLR) and is considered sufficient to capture the main differences in costs and outcomes [31]. However, a time horizon of 28 days was explored in a scenario analysis to align with the duration of the pivotal clinical trial and explore the impact of excluding long-term inputs in the results. The model adopts the NHS and PSS UK perspective, whereby all direct health effects for patients have been considered. Costs and outcomes were discounted at 3.5% per annum, in line with current National Institute for Health and Care Excellence (NICE) guidelines [32]. Total costs and QALYs were calculated based on the occurrence of events. These were accumulated over the model time horizon to calculate total costs and QALYs for the two cohorts from which incremental results and the incremental cost per QALY were determined.

### Model inputs and data sources

#### Clinical inputs

The model simulated the following clinical outcomes; treatment efficacy, progressive disease pathway, other adverse events including nephrotoxicity and septic shock, discharge to home or LTC, and mortality [29, 30, 33–35]. The model was aligned with the main source of clinical data using patient demographics at baseline from the primary analysis population mCRE-MITT of the TANGO II (Online Resource 1: Table 1) [24]. The results of TANGO II were used to inform the probability of cure, all-cause 28 day mortality, disease complications and TEAEs. Wherever data was not available from the TANGO II trial, post-hoc analysis was conducted to source probabilities for these key events. An SLR and a targeted literature review (TLR) were used to inform the remaining clinical parameters such as RRT (in hospital), chronic RRT, all-cause general mortality and mortality with chronic RRT (Table 1 and Online Resource 1: Table 2).

**Table 1:** Model inputs

Cost inputs	Frequency	Value	Source
**Drug**			
Vaborem (£)*	Per course	2,839.00	[36]
BAT (£)*	Per course	808.19	[37–44]
**Drug administration costs**			
Vaborem/BAT (£)	Per course	385.00	[45]
**Disease management costs**			
Length of stay	Hospital stay	ICU stay	
Unit cost (£)	353.12	1,847.74	[46]
% patients	83%	17%	[28]
Length of stay (days)	10.81	12.38	[28]
**Disease complication costs**			
Unit cost for LTC (£)	Annual	65,863.09	[47]
Length of clinical failure hospital stay (days)	One-off	10.81	[48]
Unit cost for clinical failure hospital stay (£)	One-off	353.12	[46]
Total cost for clinical failure hospital stay (£) (estimated as product of 2 previous inputs)	One-off	3815.47	[46, 48]
**Treatment-related adverse event costs**			
Unit cost for nephrologist referral	One-off	231.19	[49]
Unit cost for acute kidney injury	One-off	2,927.50	[50]
Unit cost for RRT (in hospital)	Per day	218.94	[51]
Length of stay for RRT (in hospital) (days)	One-off	6.00	[29]
Unit cost for CKD (exacerbation)	Annual	2,307.00	[52]
Unit cost for chronic dialysis	Annual	28,093.57	[53]
Unit cost for septic shock	Per event	2,058.48	[54]
**Clinical inputs**			
**Efficacy inputs**	Vaborem % (*n*/*N*)	BAT % (*n*/*N*)	
Clinical cure at TOC, *n*/*N* (%)	59.4% (19/32)	26.7% (4/15)	[24]
Mortality at 28 days, *n*/*N* (%)	15.6% (5/32)	33.3% (5/15)	[48]
Nephrotoxicity (renal acute failure events), *n*/*N* (%)	3.1% (1/32)	26.7% (4/15)	[48]
Septic shock, *n*/*N* (%)	3.1% (1/32)	26.7% (4/15)	[48]
**Quality of life inputs by health states**			
Health State	Value	Duration (days)	Source
**Short-term utilities**			
Hospitalisation	0.780	11.1	[48, 55]
Nephrotoxicity	0.676	118.0	[29, 56]
Post-hospitalisation (up to 28 days)	0.795	16.9	[57, 58]
**Long-term utilities**			
Chronic RRT	0.630	275.3	[55]
Home	0.840	691.4	[57]
LTC	0.640	291.4	[58]

*BAT* best available therapy; *ICU* intensive care unit; *LTC* long-term care; *RRT* renal replacement therapy; *TOC* test of care; *CKD* chronic kidney disease

^*^Refer to Fig. 3 in Online Resource 1

The UK general population mortality rate was used to inform all-cause mortality. Those on chronic RRT face an increased risk of mortality [30, 33, 34]. Based on the recommendations from the UK clinical experts, the probability of TEAEs such as nephrotoxicity and septic shock were applied to all patients who entered the model (Table 1) [26].

#### Cost and resource use

The costs included in the model comprised of treatment costs, administration costs, disease management costs, disease complication costs and TEAE costs. Where costs were not reported in 2020 GBP, they were inflated using the NHS cost inflation index [59]. NHS costs were sourced from databases such as NHS reference costs and the BNF [27, 60]. All patients received one course of treatment according to the cohort they belonged to, with posology as per BNF (Online Resource 1: Table 3) [27]. In addition to the acquisition cost of treatment, administration costs were included, which were the same for both treatment arms in the model [35]. The disease management costs comprised of hospital and intensive care unit costs and the same costs were applied in both treatment arms (Table 1) [46, 48, 61]. Patient numbers and length of stay were sourced from TANGO II trial patient-level data as shown in Table 1 [28]. Disease complication costs consisted of various sub-categories including costs associated with clinical failure and LTC [36–44, 47]. Clinical failure costs were exclusively applied to the short-term cost outcomes, while the LTC costs were applied in a longer time horizon of 5 years (Table 1) [46, 48]. The clinical failure cost comprised of antibiotic therapy costs and hospitalisation costs. As BAT was ratified to be reflective of the UK current treatment for CRE-KPC infections, it was assumed that the cost of BAT used in the model would be reflective of the costs of one additional round of antibiotic treatment. Additionally, as antibiotic therapies for CRE-KPC infections were only available through IV infusion, patients required hospitalisation for the duration of the antibiotic therapy. This additional hospitalisation cost was calculated by multiplying the average length of hospital stay from TANGO II by the cost per day of hospitalisation. The TEAE costs consisted of both renal events and septic shock [54]. Renal events consisted of nephrotoxicity, RRT (in hospital), which were only applied during the first year, and chronic RRT, applied through all five years of the model (Table 1) [29, 49–53].

#### Health-related quality of life

A health-related QoL SLR was conducted to identify papers reporting utility for UK patients with CRE-associated infections. In the absence of UK data, parameters from studies outside the UK were validated with UK clinical experts. QoL data inputs were collected for short-term and long-term events and split into the following categories: hospitalisation, nephrotoxicity, post-hospitalisation, chronic RRT and discharge to home or LTC. Utilities were used to quantify the QoL of the different events affecting patients in the short and long-term (Table 1) [29, 48, 55–58].

The model was validated by internal and external health economists. Clinical trial data underpinning the model structure and assumptions were validated by external UK clinical experts [26].

#### Sensitivity analyses

Deterministic sensitivity analyses consisted of scenario analyses to test model structural uncertainty and one-way sensitivity analysis (OWSA) to test model input parameter uncertainty. Scenario analyses were performed to assess the impact of time horizon, probability of nephrotoxicity (nephrotoxicity was defined by renal failure acute events or Class II RIFLE criteria) and the probability of 28-day mortality. Probabilistic sensitivity analysis assigned distributions to the model parameters and ran 10,000 simulations to simultaneously explore parameter uncertainty. Beta distributions were used for the percentage of male patients, the clinical probabilities and for patient health state utilities. Gamma distributions were used for age, weight, treatment cost for BAT, administration costs, disease management costs, disease complication and TEAE costs.

## Results

### Base case results

Base case results are shown in Table 2. Over a 5 year time horizon, the total incremental cost of Vaborem was £5165 (£44,606 for Vaborem vs £39,441 for BAT) and the incremental QALY was 0.366 compared to BAT (1.733 for Vaborem vs 1.367 for BAT). The incremental cost-effectiveness ratio (ICER) for Vaborem compared to BAT was £14,113 per QALY gained. The incremental LY for Vaborem compared to BAT was 0.453 (2.183 for Vaborem vs 1.730 for BAT), which was driven primarily by the higher clinical cure rate that patients on Vaborem experienced at day 28, resulting in lower mortality compared to patients on BAT.

**Table 2:** Base case results

Treatment	Total			Incremental			ICER (£) versus baseline LYs	ICER (£) versus baseline QALYs
	Costs (£)	LYs	QALYs	Costs (£)	LYs	QALYs		
BAT	39,441	1.730	1.367	–	–	–	–	
Vaborem	44,606	2.183	1.733	5,165	0.453	0.366	11,398	14,113

*BAT* best available therapy; *ICER* incremental cost-effectiveness ratio, *LY* life years, *QALYs* quality-adjusted life years

Disaggregated results demonstrated incremental benefits of Vaborem compared to BAT in terms of increased cure (59.4 vs 26.7%) and survival (11.8 vs 9.1%), and in terms of reduced incidence of nephrotoxicity (3.1 vs 26.7%) and RRT (2.3 vs 19.2%). The disaggregated costs results showed that the costs attributed to LTC (£32,094 for Vaborem vs £25,082 for BAT) and clinical failure (£1878 for Vaborem vs £3391 for BAT) were the main drivers of differences between costs in the two cohorts. QALYs disaggregated by health state showed that the largest incremental differences in QALYs were 0.310 (1.364 for Vaborem vs 1.055 for BAT), associated with discharge to home, and 0.069 (0.306 for Vaborem vs 0.236 for BAT), associated with long-term care (Table 3). This is because differences in QALYs are due to patients on Vaborem having better survival than patients on BAT in the long-term period (i.e., more than 28 days).

**Table 3:** Disaggregated costs and QALYs by health state

	Vaborem	BAT	Increment Vaborem vs BAT	% Increment vs BAT	Increment Vaborem vs BAT %
**Costs by category short-term**					
Treatment	£2,839.00	£808.19	£2,030.81	251.3%	39.3%
Administration	£385.00	£385.00	£0.00	0.0%	0.0%
Hospitalisation	£7,058.09	£7,058.09	£0.00	0.0%	0.0%
Adverse events	£64.33	£548.93	£− 484.60	− 88.3%	− 9.4%
Clinical failure	£1,878.36	£3,390.69	£− 1,512.32	− 44.6%	− 29.3%
Nephrotoxicity	£98.71	£842.32	£− 743.61	− 88.3%	− 14.4%
RRT(in hospital)	£41.22	£330.84	£− 289.62	− 87.5%	− 5.6%
**Costs by category long-term**					
RRT (in hospital)	£32.86	£221.56	£− 188.70	− 85.2%	− 3.7%
Chronic RRT	£114.57	£772.48	£− 657.91	− 85.2%	− 12.7%
LTC	£32,093.57	£25,082.52	£7,011.05	28.0%	135.7%
*Total costs (£)*	*£44,605.70*	*£39,440.62*	*£5,165.08*	*13.1%*	*100.0%*
**QALYs by category short-term**					
Hospitalisation	0.032	0.030	0.002	7.5%	0.6%
Post-hospitalisation (up to 28 days)	0.026	0.014	0.012	84.0%	3.3%
Nephrotoxicity	0.002	0.014	− 0.012	− 88.3%	− 3.3%
**QALYs by category long-term**					
Nephrotoxicity	0.000	0.002	− 0.002	− 85.2%	− 0.4%
Chronic RRT	0.002	0.016	− 0.014	− 85.2%	− 3.7%
Home	1.364	1.055	0.310	29.4%	84.6%
LTC	0.306	0.236	0.069	29.4%	19.0%
*Total QALYs*	*1.733*	*1.367*	*0.366*	*26.8%*	*100.0%*

*BAT* best available treatment; *RRT* renal replacement therapy; *LTC* long-term care; *QALY* quality-adjusted life years

### Scenario analysis results

Results were most sensitive to the time horizon. In the scenario with a time horizon of 28 days, Vaborem dominated (i.e., less costly and more effective), as it was associated with £999 less incremental costs and 0.002 more incremental QALYs. Disaggregated results demonstrated incremental benefits for Vaborem compared to BAT in terms of increased cure (59.4 vs 26.7%) and survival (84.4 vs 66.7%), but also in terms of reduced incidence of nephrotoxicity (3.1 vs 26.7%) and RRT (2.3 vs 19.2%). The disaggregated results showed that the costs attributed to treatment (£2839 for Vaborem vs £808 for BAT) and clinical failure (£1878 for Vaborem vs £3390 for BAT) were the key drivers of differences between costs in the two treatment arms.

Results were also sensitive to the probability of 28-day mortality, in the scenario where the probability was based on cure status (rather than by treatment), the ICER decreased to £12,179 per QALY gained, with an incremental total cost of £3265 (£43,050 for Vaborem vs £39,786 for BAT) and incremental QALY of 0.268 (1.652 for Vaborem vs 1.384 for BAT). Results were also sensitive to the probability of nephrotoxicity, in the scenario where the probability was defined as Class II RIFLE {rather than renal acute failure adverse events [AEs]}, the ICER increased to £18,844 per QALY gained, with an incremental total cost of £6,636 (£44,461 for Vaborem vs £37,825 for BAT) and incremental QALY of 0.352 (1.735 for Vaborem vs 1.383 for BAT).

### One-way sensitivity analysis results

**Fig. 2:**
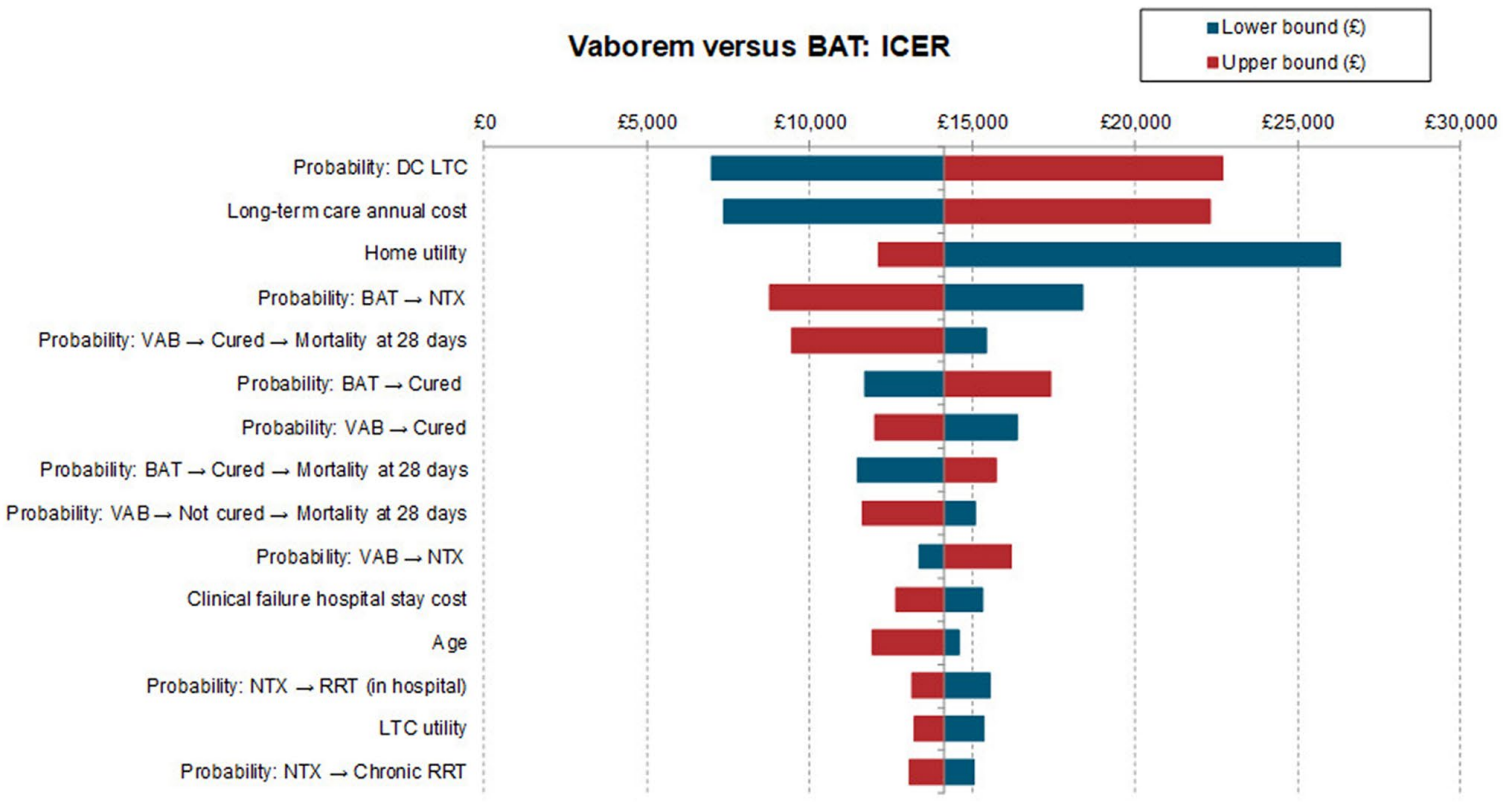
ICER tornado diagram for the one-way sensitivity analysis. Tornado diagram illustrates ICER results for the top 15 most sensitive parameters. *BAT* best available therapy; *ICER* incremental cost-effectiveness ratio; *DC LTC* discharge to long-term care; *NTX* nephrotoxicity, *VAB* Vaborem, *RRT* renal replacement therapy; *LTC* long-term care

One-way sensitivity analysis explored the level of uncertainty in the base case ICER based on the upper and lower bounds of model inputs in the form of a tornado diagram. Figure 2 depicts the results of the OWSA, showing the change in ICER across the top 15 most sensitive parameters. The ICER results were most sensitive to the probability of discharge to LTC, the annual cost of LTC and the utility of discharge to home. The lower and upper bound estimates of the probability of discharge to LTC resulted in an ICER of £6998 and £22,687, respectively. For the annual cost of LTC, the lower and upper bound estimates resulted in an ICER of £7353 and £22,320, respectively. The lower and upper bound estimates of the utility associated at home after discharge resulted in an ICER of £12,107 and £26,307, respectively. Across all parameters tested, the ICER remained below the £20,000–30,000 threshold per QALY gained.

### Probabilistic sensitivity analysis results

**Fig. 3:**
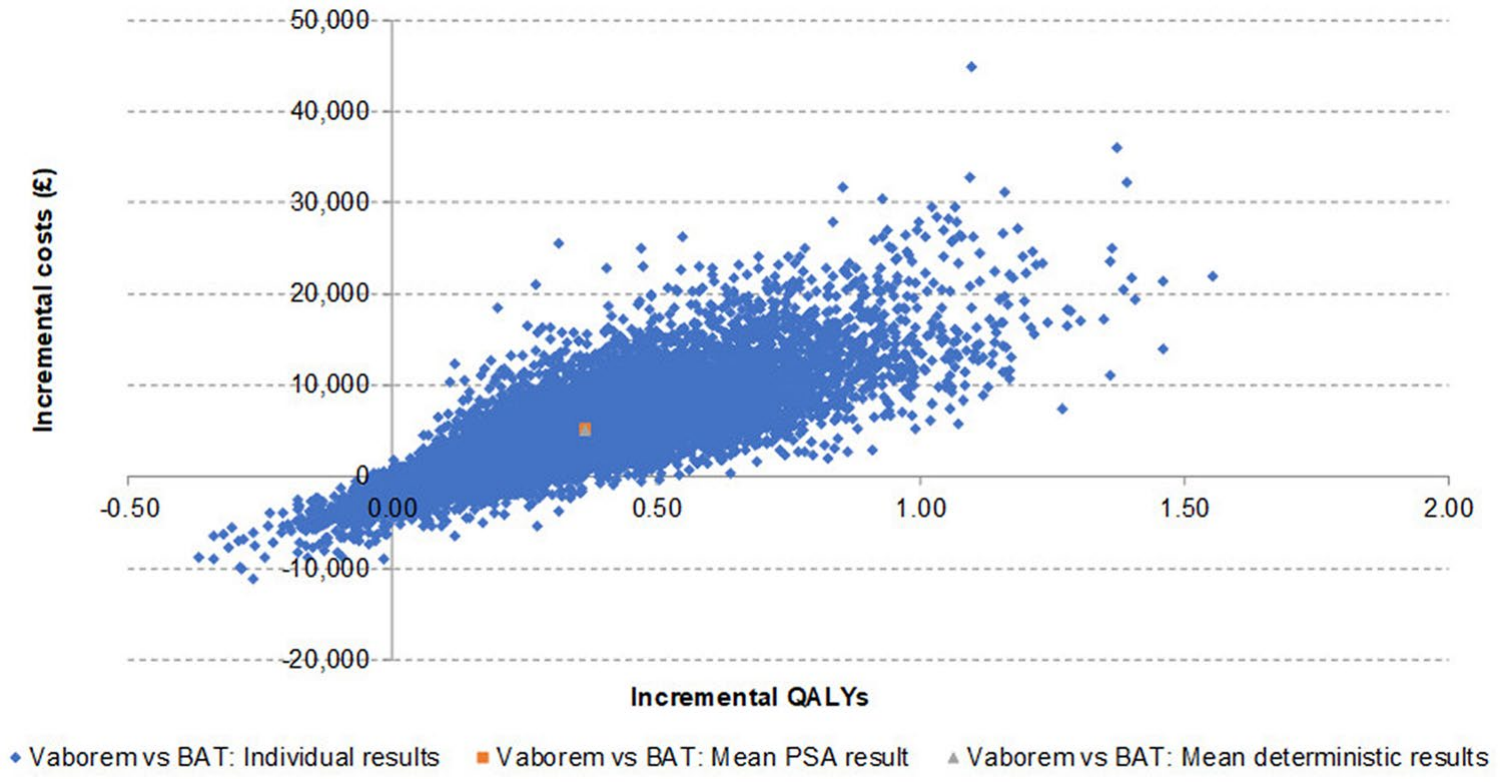
Incremental cost-effectiveness plane. The cost-effectiveness plane diagram depicts the four quadrants where X axis represents the incremental level of effectiveness of an outcome and the Y axis represents the additional total cost of implementing this outcome. *BAT* best available therapy; *PSA* probabilistic sensitivity analysis

**Fig. 4:**
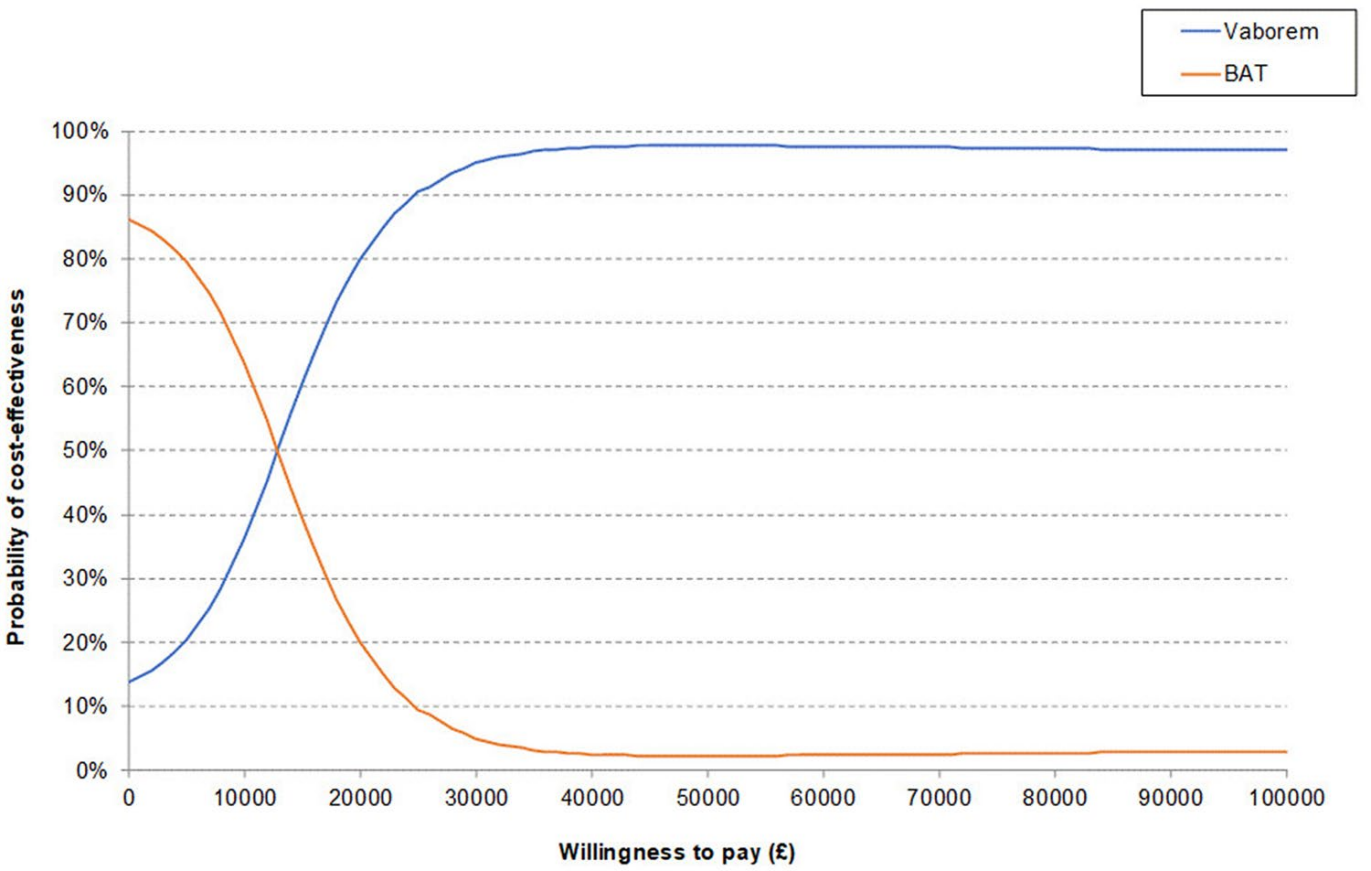
Cost-effectiveness acceptability curve. Cost-effectiveness acceptability curve illustrates the probability that Vaborem or BAT is cost-effective at various willingness to pay thresholds. *BAT* best available therapy

Mean probabilistic incremental results were recorded and illustrated in the incremental cost-effectiveness plane. In the probabilistic sensitivity analyses, the majority (86.23%) of the iterations fell in the north-east quadrant where Vaborem is more costly and more effective (Fig. 3). A CEAC illustrated the probability of Vaborem being cost-effective compared to BAT, at a range of willingness-to-pay thresholds (Fig. 4). There is a 79.9% probability of Vaborem being cost-effective at a willingness-to-pay threshold of £20,000/QALY, increasing to 94.9% at a willingness-to-pay threshold of £30,000/QALY. Therefore, Vaborem is a cost-effective treatment option for patients with CRE-KPC-associated infections at the willingness-to-pay thresholds accepted by NICE in the UK.

## Discussion

CRE-KPC infections and their increasing resistance to existing antibiotics underscore the urgent need for new effective drugs. In the last 5 years, novel drugs have been developed and to-date many are in the pipeline to fill the unmet need in this area. However, discovery, innovation and research and development of new drugs needs higher resources making the treatment more expensive compared to the old classes of drugs. Health economic studies evaluating the cost-effectiveness of these drugs are crucial to inform decision-making for their adoption in different settings. Vaborem is the first boronate β lactamase inhibitor in combination with meropenem approved against CRE-KPC infections. The base case results showed that, over a 5 year time horizon, the incremental costs of Vaborem was £5165 and the incremental QALYs was 0.366, resulting in an ICER of £14,113 per QALY gained compared to BAT. A time horizon of 28 days was utilised in a sensitivity analysis to explore the impact of excluding long-term data from the model and align with the duration of the TANGO II trial. Vaborem was dominant compared to BAT in this sensitivity analysis, due to the superior clinical cure and survival rates, therefore, validating the robustness of the results.

This is the first UK CEA study of Vaborem using data from the TANGO II study which is the first prospective clinical trial with a pathogen focus specially designed for CRE-KPC infections in severely ill patients. This model was developed based on the evidence from the TANGO II trial which demonstrated that Vaborem reduced mortality, increased cure rates and decreased nephrotoxicity compared to BAT for the treatment of CRE-KPC infections [24]. There is increasing clinical evidence for the efficacy of Vaborem from real-life settings for different indications [62, 63]. Shields et al*.* conducted an observational study which showed a 30 day survival of 90% and clinical success of 65%, in patients with 90% of infections due to CRE-KPC and who were treated with Vaborem [64].

This is the first UK study, to the authors’ knowledge, to assess the cost-effectiveness of Vaborem for the treatment of CRE-KPC infection. Recently, Simon et al*.* published their assessment on the cost-effectiveness of ceftazidime-avibactam another novel β-lactam/β-lactamase inhibitor compared to colistin-based therapy based on literature for sepsis and not specific to CRE infection in the US [31]. The strengths of this CEA compared to the study by Simon et al*.* is that, this is a pathogen-specific study that used a microbiologically enriched population (mCRE-MITT) [24]. Patient population with different sites of infection due to CRE (known as per mCRE-MITT population and suspected) were used for analysis in this study (cUTI, cIAI, HABP, VABP and bacteraemia). This study compared Vaborem with the BAT antibiotics used in the UK for the treatment of CRE-KPC infections. The model structure and key inputs were validated by clinical and health economics experts. An independent UK health economist reviewed the approach and methodology and provided suggestions for improvement in the model. Clinical trial data underpinning the model structure and assumptions were ratified by UK external clinical experts. All feedback obtained after internal and external ratification informed the final model.

In this cost-effectiveness model, the costs attributed to LTC and clinical failure were the main drivers of costs between Vaborem and BAT. This finding correlated with a higher proportion of patients treated with Vaborem who were cured from (59.4 vs 26.7%) and survived a (11.8 vs 9.1%) CRE infection compared to patients treated with BAT, as predicted by the model in the long-term. Also, the proportion of discharged patients to LTC predicted by the model was slightly higher in the Vaborem-treated arm (19.2 vs 15.2%), as more patients survived compared to BAT-treated arm. -Patients after hospitalisation can be discharged to home or LTC, and most of the patients (77.3%) in this analysis were discharged to home (Online Resource: Table 1). Therefore, given that the majority of patients spend most of their time in the ‘home discharge’ state, it explains why the QoL in this health state, is one of the key drivers of the results as shown in the OWSA. The largest incremental QALYs were associated with discharge to home (1.364) due to better survival of Vaborem-treated patients in the long-term period contributing to the cost-effectiveness of the model.

The optimal treatment of infections due to CRE-KPC organisms is uncertain because of the observational nature of most of the studies on the few available antibiotic options, often used as combinations. These are associated with increased toxicity, suboptimal PK/PD profile leading to failure and emergence of resistance and higher costs, leading to increased healthcare expenditure. The proportion of Vaborem-treated patients suffering from any AEs, particularly nephrotoxicity, were fewer compared to BAT-treated patients (3.1 vs 26.7%) and therefore the corresponding costs associated with nephrotoxicity were lower in Vaborem-treated patients.

Pathogen-focused or resistance-focused clinical trials are crucial to accurately determine the efficacy of new treatments, yet enrolment is exceedingly difficult due to the high-risk population. These difficulties are typical of the “ultra-orphan” world of antimicrobial development, where new treatments are needed. Due to the decrease in mortality rates, higher clinical benefit and lower renal events seen in the TANGO II study, the Data Safety Monitoring Board, based on a risk/benefit analysis, decided to stop further randomisation to the BAT arm and the study was terminated earlier. In this trial, 77 patients were enrolled and took 2.5 years to enrol the last subject. However, TANGO II study design allowed the enrolment of subjects who would typically be excluded from pivotal clinical trials, such as those with many comorbidities as well as an immunocompromised state.

To model the complex clinical pathway of CRE infections, it was necessary to apply some assumptions. A decision tree structure was used to capture important costs and consequences associated with CRE-KPC infections that focused on cure, survival and long-term effects of toxicity. For clinical effectiveness, cure, 28 day mortality, clinical failure, patients in LTC, nephrotoxicity, septic shock, RRT (in hospital) and chronic RRT inputs were used. Relevant cost categories included were treatment costs, administration costs, disease management costs, TEAE costs and disease complication costs. Utility approach was used to capture the health-related QoL impact of treatment for CRE-KPC infections. All assumptions are summarised in the Online Resource: Table 4.

The study sample size could be deemed small as it comprised the mCRE-MITT population from the TANGO II trial. However, the majority of the pivotal noninferiority trials for antibiotics against multidrug-resistant pathogens, with a microbiologically enriched population have an even smaller sample size of multidrug-resistant isolates. The small sample size is also a reflection of the difficulties linked to this specific epidemiological setting, similar to rare diseases and barriers in recruiting these patients with high screening failure rates. The patients in the TANGO-II trial were enrolled in a timeframe of 2.5 years, demonstrating issues in conducting pathogen-driven trials and the reluctance of very unwell patients and their relatives to participate in a clinical trial that uses an experimental, new antimicrobial. Though the number of patients enrolled are low, all had CRE (known or suspected infections) and isolates were mostly KPC with high minimum inhibitory concentration (MIC) for meropenem, consistent with the clinical setting. No clinically relevant increases in MICs occurred during treatment with Vaborem. It should be noted also that the open label design of TANGO II was chosen to enable investigators to treat these critically unwell patients in a timely manner, with the most efficacious therapy. Another limitation is that the TANGO II has a short-term follow-up (28 days), although this is a common follow-up in antibiotics trials due to the short half-life of the drugs and short treatment duration. Some assumptions based on published literature and key opinion leader (KOL) opinion were needed to simulate results over a longer time horizon of five years. Also, potential antimicrobial resistance arising due to antibiotic use and its costs were not considered in this model. It is estimated that the impact of antimicrobial resistance on the cost-effectiveness of Vaborem would be minimal as Vaborem has a low propensity for resistance selection and is active against strains producing KPC mutants resistant to ceftazidime-avibactam and colistin [25]. However, this can be an area of research in future studies.

## Conclusion

Owing to a limited cost impact and an improvement in patients’ QoL, Vaborem can be considered a cost-effective treatment option compared to BAT for adult patients with CRE-KPC infections in the UK.

## Supplementary Information

Below is the link to the electronic supplementary material.

Supplementary file1 (DOCX 138 KB)

## Data Availability

All data generated or analysed during this study is included in this published article (and its supplementary information files).
