# Supplementary material for: Cost-effectiveness analysis of vaborem for the treatment of carbapenem-resistant Enterobacteriaceae-Klebsiella pneumoniae carbapenemase (CRE-KPC) infections in the UK
Source: Eur J Health Econ. 2021 Sep 21;23(3):537–49. doi: 10.1007/s10198-021-01375-0 (PMC8453464; doi:10.1007/s10198-021-01375-0)
Supplement: Supplementary file 1 — Supplementary file1 (DOCX 138 KB) [file 10198_2021_1375_MOESM1_ESM.docx]

**SUPPLEMENTARY INFORMATION**

Title: Cost-effectiveness analysis of Vaborem for the treatment of Carbapenem-Resistant Enterobacteriaceae - *Klebsiella Pneumoniae* Carbapenemase (CRE-KPC) infections in the UK

Journal name: The European Journal of Health Economics

Author names: Ioanna Vlachaki^1^, Daniela Zinzi^2^, Edel Falla^3^, Theo Mantopoulos^4^, Holly Guy^5^, Jasimran Jandu^5^ & Andrew Dodgson^6^

Affiliations:

^1^ Menarini Ricerche Spa, Athens, Greece

^2^ Menarini Ricerche SpA, Florence, Italy

^3^Real World Solutions, IQVIA ltd, London, United Kingdom

^4^ Real World Solutions, IQVIA ltd, Athens, Greece

^5^ FIECON Ltd, St Albans, United Kingdom

^6^ Public Health England, Manchester, UK.

Corresponding author e-mail address: [edel.falla@iqvia.com](mailto:edel.falla@iqvia.com)

Figure 1: Sub-tree 1 model structure


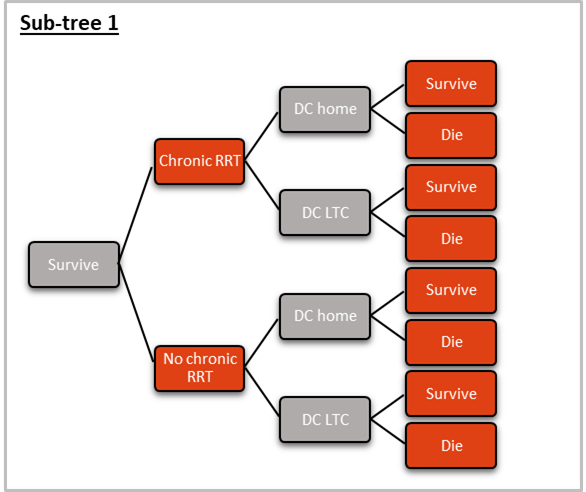


^DC, discharged; RRT, renal replacement therapy, LTC, long-term care^

Figure 2: Sub-tree 2 model structure


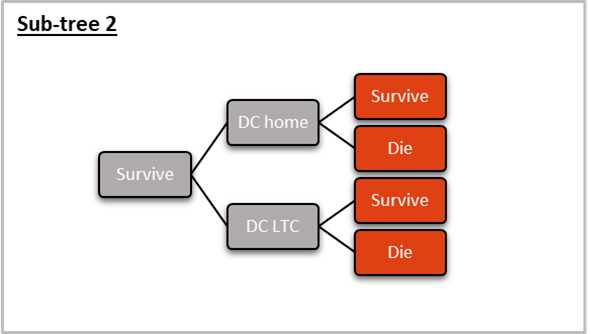


^DC, discharged; LTC, long-term care^

Table 1: Baseline demographics of cohort contributing to the model based on the mCRE-MITT population of TANGO II

| **Parameter at baseline** | **Base Case** | **Source** |
| --- | --- | --- |
| Mean age (years) | 62.5 | [24] |
| Percentage male | 51% | [24] |
| Mean weight (kg) | 76.11 | [65] |
| CCI score ≤2, n/N (%) | 5/47 (10.6%) | [24] |
| CCI score 3, n/N (%) | 2/47 (4.3%) | [65] |
| CCI score 4, n/N (%) | 3/47 (6.4%) | [65] |
| CCI score 5, n/N (%) | 12/47 (25.5%) | [24] |
| CCI score ≥6, n/N (%) | 25/47 (53.2%) | [24] |

^BAT, best available therapy; CCI, Charlson comorbidity index; kg, kilogram^

Table 2: Summary on disease pathway & mortality inputs

| **Disease pathway** | **Value** | **Source** |
| --- | --- | --- |
| RRT (in hospital) - Renal acute failure events | 72.0% | [29] |
| Chronic RRT | 12.8% | [29] |
| Discharged to home | 77.3% | [40] |
| Discharged to LTC | 22.7% | [40] |
| **Mortality inputs** | | |
| All-cause mortality adjusted for CCI | | |
| Year 1 | 31.1% | [24, 30, 33] |
| Year 2 | 31.8% |  |
| Year 3 | 32.4% |  |
| Year 4 | 33.1% |  |
| Year 5 | 33.9% |  |
| Chronic RRT adjusted for CCI | | |
| Year 1 | 48.9% | [24, 33, 34] |
| Year 2 | 56.1% |  |
| Year 3 | 60.1% |  |
| Year 4 | 74.3% |  |
| Year 5 | 77.0% |  |

^RRT, renal replacement therapy; LTC, long-term care; CCI, Charlson comorbidity index^

Table 3: Summary of treatment costs for Vaborem and BAT

| **Drug class** | **Drug^*^** | **% of treatment combination** | **Formulation per vial (mg)** | **% therapy usage** | **Unit cost per vial (£)** | **Cost per drug course (£)** |
| --- | --- | --- | --- | --- | --- | --- |
| Vaborem | Vaborem | 100% | 1,000 | 100% | 55.67 | 2,839.0 |
| **BAT** | | | | | | |
| *Monotherapy* | | | | | |  |
| Aminoglycoside | Amikacin | 0% | 500 | 7% | 12.00 | 808.2 |
|  | Gentamicin | 100% | 80 |  | 2.01 |  |
| Carbapenem | Ertapenem | 100% | 1,000 | 7% | 31.65 |  |
|  | Meropenem | 100% | 500 |  | 8.30 |  |
| Ceftazidime-avibactam | Ceftazidime | 100% | 2,000 | 7% | 85.70 |  |
|  | Avibactam | 100% | 500 |  |  |  |
| Polymyxin/colistin | Colistin | 0% | 2 million units | 7% | 3.24 |  |
|  | Polymyxin | 100% | 500,000 polymyxin units |  | 1.80 |  |
| *Dual therapy* | | | | | |  |
| Carbapenem + aminoglycoside | Amikacin | 0% | 500 | 7% | 12.00 |  |
|  | Gentamicin | 100% | 80 |  | 2.01 |  |
|  | Ertapenem | 0% | 1,000 |  | 31.65 |  |
|  | Meropenem | 100% | 500 |  | 8.30 |  |
| Carbapenem + polymyxin/colistin | Ertapenem | 100% | 1,000 | 7% | 31.65 |  |
|  | Meropenem | 100% | 500 |  | 8.30 |  |
|  | Colistin | 100% | 2 million units |  | 3.24 |  |
|  | Polymyxin | 0% | 500,000 polymyxin units |  | 1.80 |  |
| Carbapenem + tigecycline | Ertapenem | 0% | 1,000 | 13% | 31.65 |  |
|  | Meropenem | 100% | 500 |  | 8.30 |  |
|  | Tigecycline | 100% | 50 |  | 29.08 |  |
| Polymyxin/colistin + aminoglycoside | Amikacin | 67% | 500 | 20% | 12.00 |  |
|  | Gentamicin | 33% | 80 |  | 2.01 |  |
|  | Colistin | 100% | 2 million units |  | 3.24 |  |
|  | Polymyxin | 0% | 500,000 |  | 1.80 |  |
| *Triple therapy* | | | | | |  |
| Carbapenem + polymyxin/colistin + tigecycline | Ertapenem | 0% | 1,000 | 7% | 31.65 |  |
|  | Meropenem | 100% | 500 |  | 8.30 |  |
|  | Tigecycline | 100% | 50 |  | 29.08 |  |
|  | Colistin | 100% | 2 million units |  | 3.24 |  |
|  | Polymyxin | 0% | 500,000 polymyxin units |  | 1.80 |  |
| Carbapenem + polymyxin/colistin + ceftazidime-avibactam | Ertapenem | 0% | 1,000 | 7% | 31.65 |  |
|  | Meropenem | 100% | 500 |  | 8.30 |  |
|  | Ceftazidime | 100% | 2,000 |  | 85.70 |  |
|  | Avibactam | 100% | 500 |  |  |  |
|  | Colistin | 0% | 2 million units |  | 3.24 |  |
|  | Polymyxin | 100% | 500,000 polymyxin units |  | 1.80 |  |
| *Four drugs or more* | | | | | |  |
| Carbapenem + polymyxin/colistin + aminoglycoside + tigecycline | Ertapenem | 0% | 1,000 | 13% | 31.65 |  |
|  | Meropenem | 100% | 500 |  | 8.30 |  |
|  | Tigecycline | 100% | 50 |  | 29.08 |  |
|  | Colistin | 100% | 2 million units |  | 3.24 |  |
|  | Polymyxin | 0% | 500,000 polymyxin units |  | 1.80 |  |
|  | Amikacin | 0% | 500 |  | 12.00 |  |
|  | Gentamicin | 100% | 80 |  | 2.01 |  |

**^*^** ^Dosing as per British National Formulary [27]. Sources: Vaborem [42], BAT [27, 43-50]^

Table 4: Model assumptions and justifications

| **Assumption** | **Justification** |
| --- | --- |
| **Time horizon** | |
| A maximum lifetime horizon of 5 years is used. | A 5-year time horizon is appropriate to allow the main differences in costs and outcomes to be captured. The mean age of patients at baseline is 62.5 years and following all-cause mortality adjusted for CCI at baseline and the additional risk of mortality due to chronic RRT there are 10.3% and 8.0% of patients alive at 5 years in the Vaborem and BAT treatment arms, respectively. Further extending the time horizon to allow all patients to have died, would carry increased uncertainty. In addition, the 5-year time horizon has been used in a previous cost-utility study identified in an economic evaluation SLR. |
| **Model structure** | |
| The important costs and consequences associated with CRE-KPC infections can be captured through a decision tree structure focusing on cure, survival and the long-term effects of toxicity. | Infections from CRE-KPC are associated with poor survival and most frequently affect vulnerable individuals in long-term acute healthcare settings. Existing treatments incur high risk of nephrotoxicity thus translating into high healthcare costs and poor clinical outcomes in the short and long-term. Treatment with Vaborem has shown improvements in the rates of toxicity, cure status and survival. Modelling these outcomes upon comparison of the treatments demonstrates the value of Vaborem in terms of costs and consequences.  A decision tree structure works well with the occurrence of events and timepoints of data collected within TANGO II allowing the model to simulate short-term events and longer-term events of different durations. |
| **Clinical effectiveness** | |
| Day-28 mortality source. | UK clinical experts advised that the base case model should be informed by the randomised controlled comparison of Vaborem vs BAT, as per the TANGO II trial. A comparison of mortality by cure status may be biased as the sicker, through existing comorbidities or the infection itself, patients may be less likely to be cured. Other studies, including ceftazidime-avibactam vs BAT, have come to conclusions of a similar overall effect and magnitude. |
| Nephrotoxicity definition. | Nephrotoxicity defined using renal AEs formed the base case model given that it is more representative of the patient population. |
| Patients who have been discharged to LTC remain so until they leave the model. | Patients are expected to remain in LTC once they have been discharged for the remainder of the model given the age of the patient population at baseline and anticipated comorbidities. This was ratified by UK clinical expert opinion. |
| Patients who are on chronic RRT remain so until they leave the model. | Patients are expected to remain on chronic RRT (acquired at day 90) until they die or the model ends. The patient population would not be considered for other CKD treatments such as kidney transplant due to their poor survival rates based on underlying comorbidities. This was ratified by UK clinical expert opinion. |
| All-cause mortality was adjusted by the CCI score at baseline in TANGO II. | It is anticipated that the licensed population for Vaborem will have several underlying comorbidities similar to the baseline demographics of the mCRE-MITT population observed in TANGO II. Given the severity of such infections and the setting by which they are acquired, UK clinical experts ratified that all-cause mortality should be adjusted in this way, to account for the increased risk of mortality faced by those eligible for treatment with Vaborem. |
| **Cost and resource use** | |
| Relevant cost categories include treatment costs, administration costs, disease management costs (including length and type of hospital stay), treatment-emergent adverse event costs (including NTX and septic shock), and disease complication costs (including LTC and clinical failure). | Published literature and expert opinion. |
| Patients who are not clinically cured receive another round of antibiotic treatment and hospitalisation. | UK clinical expert opinion confirmed that if patients are not cured at TOC, in practice they would receive another round of antibiotics (varying drugs, dosing or regimen) and would stay in hospital for the duration of the treatment. |
| **Quality of life inputs** | |
| Utilities adequately capture the health-related QoL impact of treatment for CRE-KPC infections. | The utility approach is accepted and recommended in HTA submissions. The duration for each event was sourced from literature and ratified by UK clinical expert opinion. |

^CCI, Charlson comorbidity index; BAT, best available treatment; SLR, systematic literature review; CRE-KPC, Carbapenem-Resistant Enterobacteriaceae - Klebsiella Pneumoniae Carbapenemases; UK, United Kingdom; AEs, adverse events; LTC, long-term care; RRT, renal replacement therapy; CKD, Chronic kidney disease; QoL, quality of life; HTA, health technology assessment; NTX, nephrotoxicity; TOC, test of care; CKD, chronic kidney disease.^
